# Checkmate awaiting strategy: unlocking the potential of chimeric antigen receptor T-cell therapy in uveal melanoma

**DOI:** 10.3389/fonc.2025.1555842

**Published:** 2025-02-18

**Authors:** Ahmed H. Al Sharie, Samah O. Al-Omari, Abdelwahab Aleshawi, Rami Al-Dwairi, Osama Al Deyabat

**Affiliations:** ^1^ Department of Pathology and Microbiology, Faculty of Medicine, Jordan University of Science and Technology, Irbid, Jordan; ^2^ Department of Internal Medicine, Faculty of Medicine, Jordan University of Science and Technology, Irbid, Jordan; ^3^ Division of Ophthalmology, Department of Special Surgery, Faculty of Medicine, Jordan University of Science and Technology, Irbid, Jordan; ^4^ University of Illinois, Peoria, IL, United States; ^5^ The Hashemite University, Zarqa, Jordan

**Keywords:** uveal melanoma, chimeric antigen receptor t cells therapy, CAR-T, preclinical studies, clinical trials, and immunotherapy

## 1 Introduction

Uveal melanoma (UM) is the most common primary intraocular malignancy in adults with unique genomic and phenotypic features in comparison to its cutaneous counterpart (1). UM is classified based on the anatomical location it originates from, including choroid, ciliary body, and the iris (1). Risk factors linked to UM includes the presence of atypical cutaneous nevi, light eye color, fair skin color, cutaneous freckles, occupational exposure to irritants, and many others (2). The genetic landscape of UM is characterized by chromosomal rearrangements involving chromosomes 1, 3, 6, and 8, along with a broad spectrum of oncogenic somatic mutations. Notably, mutations in genes such as *BAP1*, *EIF1AX*, *GNA11*, *GNAQ*, and *SF3B1* are frequently observed (3). Although several treatment modalities have been investigated over the past decade including local and systemic interventions, the prognosis of UM remains poor (4, 5). Local control could be achieved but ultimately, 40% of patients will develop distant metastasis, commonly to the liver (6). Recent advances in understanding the tumorigenic process and immune microenvironment of UM have enhanced prognostic assessments and paved the way for promising novel therapeutic approaches. However, progress in the development of therapeutic approaches has been slow and limited.

## 2 Chimeric antigen receptor-T cells therapy in UM

Recently, CAR-T cells therapy has revolutionized the field of anti-cancer immunotherapy. Such success story with remarkable efficacy and durable clinical responses especially in hematological malignancies has shed the light on the role of CAR-T in other malignancies (7). The CAR-T cells therapy incorporates synthetic receptors into lymphocytes, redirecting them to recognize cells expressing a specific target antigen. The process starts with careful patient evaluation and selection followed by leukapheresis. Isolated and purified lymphocytes are genetically engineered to express chimeric antigen receptors (CARs). After lymphodepletion, the CAR-T cells are infused and a clinical response is then monitored (7). The significant response rates observed with CAR-T cell therapies have led to the US Food and Drug Administration (FDA) approval of seven treatments, with many more currently being extensively studied in both basic and clinical research. An emerging area of research is the use of CAR-T cell therapy in cutaneous melanoma, with numerous preclinical studies and ongoing clinical trials exploring its potential mainly targeting c-Met, CD70, GD2, VEGFR2 (8). While CAR-T cell therapy has garnered significant attention and extensive research in the treatment of cutaneous melanomas, similar focus and investigation are notably scarce in the case of UM, where the application of CAR-T therapy remains largely underexplored.

Exploring the landscape of ongoing research on CAR-T therapy in UM reveals that it is primary laboratory investigations, with minimal clinical testing conducted thus far, and any existing studies are often part of broader cutaneous melanoma trials. Forsberg et al. have investigated the utilization of HER2 CAR-T cells to eradicate UM *in-vitro* and *in-vivo* (9). The latter was conducted in a novel humanized mouse model using NOD/SCID IL-2 receptor gamma knockout mice that are transgenic for human IL-2. It was found that melanoma exhibits a variable expressivity for HER2 and the HER2 CAR-T cells were able to kill tumor cells *in-vitro* and *in-vivo*. Notably, HER2 CAR-T cells demonstrated significantly greater efficacy against UM cells from non-responders compared to autologous tumor-infiltrating lymphocyte (TIL) therapy. The aforementioned findings were target-specific; confirmed by the efficacy loss upon CRISPR/Cas9-mediated disruption of HER2 in the melanoma cells. Another targeted antigen is tyrosinase related protein 1 (TYRP1). Jilani et al. have engineered a CAR-T cell therapy targeting TYRP1 against cutaneous and uveal melanomas that are unresponsive to immune checkpoint blockade (10). The research team has highlighted the significant TYRP1 overexpression in cancer cells and demonstrated remarkable efficacy both *in vitro* and *in-vivo* murine/patient-derived melanoma models. Hackett et al. observed similar outcomes, showing that TYRP1-targeted CAR T cells activate antigen-specific responses and exhibit cytotoxic activity against human melanomas both *in vitro* and *in-vivo*, irrespective of MHC alleles and expression (11). Additionally, the toxicity to pigmented normal tissues, which was seen with TYRP1-targeted TCR-expressing T lymphocytes, was not present with the TYRP1-targeted CAR T cells. B7-H3 is a highly expressed antigen in UM representing a promising target of CAR-T cell therapy. The innovative approach proposed and applied by Ventin et al. engineered B7-H3 CAR-T cells coupled with inducible caspase-9 (iCas9) suicide gene. iCas9.B7-H3 CAR-T cells showed significant antitumor response both *in vitro* and *in vivo*; using UM experimental models of liver metastases (12). The durable outcome was comparable to B7-H3 blockage with humanized monoclonal antibody. CAR-T cell therapy targeting HER2, TYRP1, and B7-H3 showed remarkable efficacy and safety profiles that supports the translational research into a phase I clinical trial.

To the best of our knowledge, and through review of multiple clinical trials registries, two clinical trials are currently investigating CAR-T cell therapy in UM patients, although these trials are not specifically designed for UM patients. The GAIL-N trial (ClinicalTrials.gov identifier: NCT03635632) aims to improve cancer treatment for patients with neuroblastoma, sarcoma, UM, breast cancer, or other cancers expressing GD2. Such therapy has been equipped with C7R gene which provides a constant cytokine supply maintaining a longer survival time for transfused CAR-T cells. The GAIL-N trial is a non-randomized, open label, Phase I, single group assignment trials stated on the 23^rd^ of April, 2019 and currently is active and not recruiting with an estimated number of 94 participants. The trial is being sponsored by Baylor College of Medicine and no results are available up to this point of time. Another clinical trial (ClinicalTrials.gov identifier: NCT04119024) investigates the safety of IL-13Rα2-targeting CAR-T cell therapy in stage IIIC or IV melanoma or metastatic solid tumors. IL-13Rα2-targeting CAR-T cells are infused after chemotherapy conditioning regimen and the endpoints are to detect the maximum tolerated dose, adverse events profile and anti-tumor responses. The trial is an open-label, Phase I, single group assignment trial started in the 27^th^ of November, 2019 and is currently recruiting eligible participants. The trial is sponsored by Jonsson Comprehensive Cancer Center.

## 3 Discussion

CAR-T cell therapy in UM exhibits a promising preclinical finding that should be augmented with comprehensive translation research. The future of CAR-T therapy in UM hinges on continued innovative research, collaborative efforts between preclinical and clinical researchers, and the development of tailored strategies that overcome the unique challenges. The challenges are mainly focused on low specificity and tumor antigen escape, improving the safety profile eliminating CAR-T-related toxicities, overcoming the immunosuppressive nature of the tumor microenvironment, and the expense and treatment economics. With ongoing advancements, CAR-T therapy holds the potential to become an effective treatment option for UM, offering new hope for UM patients.
